# Influence of Microstructure Evolution on Tribological and Corrosion Performances of QPQ-Treated 40Cr Steel

**DOI:** 10.3390/ma19081557

**Published:** 2026-04-13

**Authors:** Jingtao Yang, Chengyuan Ni, Sen Feng, Chengdong Xia, Minghua Yin

**Affiliations:** 1 College of Mechanical Engineering, Zhejiang University of Technology, Hangzhou 310023, China; yangjt340@163.com; 2 Zhejiang Provincial Key Laboratory of Intelligent Manufacturing for Aerodynamic Equipment, Quzhou 324000, China; 3 Zhejiang Zhongcheng Sliding Bearing Technology Co., Ltd., Quzhou 324000, China

**Keywords:** 40Cr, QPQ treatment, microstructure evolution, tribological performance, corrosion resistance

## Abstract

Quench–polish–quench (QPQ) of 40Cr steel was performed to improve its tribological properties and corrosion resistance, thereby enhancing the service performance of components such as gears and bearings. The 40Cr steel was treated by QPQ at 580 °C and 620 °C for 90 or 120 min. Optical microscopy (OM, Sunny Group, Ningbo, China), scanning electron microscopy (SEM, Hitachi, Tokyo, Japan), and X-ray diffraction (XRD Rigaku Corporation, Tokyo, Japan) were used to characterise the microstructure and phase constitution. Ball-on-disk tribometry, electrochemical tests, and salt spray tests in 3.5 wt.% NaCl evaluated surface performance. At 580 °C, a composite structure of Fe_3_O_4_ and ε-Fe_2−3_N formed on the surface. When the temperature rose to 620 °C, ε-Fe_2–3_N gradually transformed into γ′-Fe_4_N. Within the scope of this study, the diffusion layer depth exhibits an approximately linear relationship with increasing processing temperature and holding time, and the surface hardness is 67–112% higher than that of the untreated sample. After QPQ treatment, the wear mechanism changed from adhesive wear to abrasive wear. However, under the treatment conditions of 620 °C × 120 min, brittle surface spalling increased roughness, thereby increasing the coefficient of friction. As treatment time increases, nitrogen atoms continue to diffuse outward as Fe_2_N transforms to the γ′ phase. This increases the composite layer’s porosity and decreases its corrosion resistance. The best corrosion resistance was observed at 580 °C for 120 min, with a corrosion potential of −0.4325 V, corrosion current density of 1.80 × 10^−6^ A·cm^−2^, and polarisation resistance of 24,500 Ω. Corrosion performance depends on overall surface integrity. Porosity morphology strongly influences this property. For 40Cr steel, the results show that surface properties are primarily determined by the quality of the compound layer’s microstructure. Specifically, density, phase-composition stability, and defect control are more important than the commonly held view of layer thickness.

## 1. Introduction

Harsh service environments, such as lubricating oil splashes, dust intrusion, and heavy rain, shorten the service life of construction machinery. They also reduce operational safety. In such environments, wear and corrosion are the most common causes of failure in mechanical parts like gears and bearings. These issues account for 60–80% of failures and usually occur on the surface or near the surface of the parts [1]. Therefore, in such environments, the surface integrity of key components, such as gears and bearings, is critical. It largely determines transmission accuracy, operational reliability, and service life [2]. These scenarios impose stringent demands on material performance. Materials must have high strength, impact toughness, fatigue, and wear resistance to withstand combined loads, including vibration, impact, and torsion. In addition, they require outstanding corrosion resistance to prevent entry of corrosive media such as mud, dust, and polluted lubricants [3,4,5]. Accordingly, surface-strengthening technologies such as carburising, nitriding, laser treatment, induction hardening, and shot peening are widely used in engineering practice [6,7]. These technologies improve surface performance by modifying near-surface composition, microstructure, and residual stress, thereby extending component life under severe conditions [8]. Because these methods differ in strengthening mechanisms, hardened-layer depth, heat-treatment temperature, and dimensional stability, their suitability depends on component type and service conditions.

Carburizing and nitriding enhance the surface performance of gear components and drive shafts. Recently, research has shifted from single to composite strengthening strategies, combining techniques like wet shot peening, isothermal quenching, cyclic carburizing, and cryogenic treatment to synergistically improve surface hardness, fatigue resistance, and tribological properties [9,10,11,12]. However, carburizing also has some limitations, such as high processing temperatures, long processing times, easy dimensional deformation, and residual stress concentration [13]. In contrast, nitriding is usually carried out at lower temperatures, resulting in smaller dimensional distortions; at the same time, it can significantly improve surface properties by forming nitrogen-rich structures [14,15]. Nitriding temperature is a key parameter [16]. As the temperature rises, the hardness of the nitrided layer and thermal deformation increase, while surface roughness changes only slightly. Laser nitriding provides better control over layer thickness, hardness, and wear resistance, and is used to strengthen titanium alloys [17,18,19,20,21,22]. However, none of the above processes can meet the requirements of low deformation, short cycle time, and low cost. In contrast, QPQ is a mixed surface processing method that combines nitrocarburizing (or nitriding) and oxidation in a salt bath. Compared to gas or plasma nitriding, it can form a compact, multi-component N–C–O modified layer on the material surface. With a very small shape change, QPQ can simultaneously improve surface hardness, wear resistance, and corrosion resistance. As a result, it has been widely applied [23,24,25].

The microstructure and performance of QPQ-treated layers are determined by treatment temperature and holding time. Proper processing conditions ensure the formation of a compact compound layer, maximising hardness, tribological performance, and corrosion resistance. In contrast, excessive temperature or prolonged treatment consistently increases brittleness, induces pore formation, and reduces the structural stability of the modified layer [26,27]. Similar trends have been reported for several steels, including 316 L stainless steel, 39NiCrMo3 structural steel, and 45 steel, indicating that QPQ can effectively enhance surface performance when the process window is properly controlled [26,28,29,30,31].

For low-alloy steels, evidence indicates considerable potential for QPQ surface modification. Yu et al. found that treating 40Cr steel with QPQ at 570 °C achieves the same hardness as ion nitriding but at a lower cost [32]. Chen et al. demonstrated that QPQ treatment improved wear, corrosion resistance, and fatigue performance in AISI 4140 steel [33]. Nevertheless, current research on QPQ treatment of 40Cr steel mainly focuses on process conditions below 590 °C, with relatively few studies on high-temperature treatment above 590 °C [32,34]. Although Zhen studied the wear and corrosion resistance of 40Cr steel treated at 630 °C, he did not investigate its microstructure changes in depth [35]. Therefore, the evolution of the surface microstructure of 40Cr steel under high temperature conditions and its influence on wear resistance and corrosion resistance still need to be systematically studied [35,36].

The corrosion resistance of 40Cr steel is limited by its medium-carbon matrix and relatively low chromium content, making it susceptible to general and localized corrosion in humid or chloride-rich environments. Over time, wear and corrosion accelerate each other, leading to dimensional inaccuracies and a gradual performance decline. Thus, enhancing surface properties is crucial. This study examined 40Cr steel with varying QPQ treatments to compare the microstructure, phase composition, and defect characteristics of the modified layer under different conditions. The goal was to establish how these features relate to hardness, friction, and corrosion resistance. The work also aims to elucidate how high-temperature QPQ treatment affects phase evolution and layer integrity, and explore their relationship with performance, thereby providing a clearer basis for process optimization.

## 2. Materials and Methods

Commercial 40Cr steel bars (Ø15 mm) were used in this study. The chemical composition (wt.%) was C 0.40, Cr 0.91, Si 0.27, P and S ≤ 0.02 each, with Fe as the balance. After quenching at 840 °C and tempering at 600 °C, the sample underwent QPQ surface modification. The QPQ processing route is summarized in Table 1. Based on the Fe–N phase diagram and previous QPQ studies, 590 °C marks a critical temperature; around this point, the phase composition and stability of the compound layer may change significantly. Thus, 580 °C and 620 °C were chosen as representative conditions, each just below and above this critical region. 580 °C reflects a conventional QPQ treatment, while 620 °C assesses a high-temperature QPQ treatment. Holding times of 90 and 120 min represent medium and extended durations, respectively.

The nitriding salt (a mixture of potassium/sodium carbonates and cyanates) and the oxidizing salt (a mixture of alkali-metal nitrates and carbonates) were supplied by Sichuan Hanke Surface Metal Materials Co., Ltd (Chengdu, China). The cyanate concentration should be maintained at 32–34 wt.%. Subsequently, disk samples (Ø15 mm × 5 mm) were fabricated by wire electrical discharge machining for microstructural characterization, hardness testing, case-depth measurement, tribological testing, and electrochemical measurements. Uncut bars were used for salt-spray testing. Unless otherwise noted, all experiments were conducted at room temperature.

After cold mounting, the specimens were gradually rubbed with silicon carbide papers of grit sizes ranging from 180 to 2000. Next, diamond pastes of grades W_2.5_ and W_0.5_ were used to polish them until no scratches remained on the surface. Subsequently, the polished surfaces were placed in a 4% nital solution for etching. An RX50M optical microscope (Sunny Group, Ningbo, China) and an SU8000 scanning electron microscope (Hitachi, Tokyo, Japan) were applied to analyze the microstructure and element distribution.

The defect rate in the composite layer was estimated using ImageJ software (v1.54f). For each sample, the compound layer region was cropped from a cross-sectional scanning electron microscope (SEM) image at 1000× magnification. All cross-sectional SEM images were converted to grayscale. They were then binarized using ImageJ’s thresholding function. The same threshold range (0–88, default mode) was used to distinguish visible defects, such as pores, from the surrounding matrix. The area fraction of the thresholded region was the defect rate. Each sample was measured three times.

X-ray diffraction analysis was performed using an Ultima IV diffractometer (Rigaku Corporation, Tokyo, Japan) equipped with a copper target to determine the phase composition of the sample surface before and after QPQ treatment. The XRD scan range was 2θ = 10° to 80°, with a scan rate of 5°/min.

Surface and cross-sectional hardness profiles were measured using an HVC-5D1 Vickers hardness tester (Beijing Time Huibo Technology Co., Ltd, Beijing, China). Before testing, each sample was ground with 1200 and 1500-grit silicon carbide papers, followed by light polishing to remove surface oxides. For every sample, six indentations were made during surface hardness testing. Cross-sectional samples for hardness profiling were prepared similarly to metallographic samples, but were not etched. Indentations were made under a 0.2 kgf load (HV_0.2_) with a 10 s dwell time, and three measurements were performed for each sample. To minimize interactions between stress fields, the spacing between adjacent indents was at least three times the diagonal length of the initial indent.

Dry-sliding tribological performance was evaluated at room temperature using an MXS-01 ball-on-disk tribometer (A&T Rongqian Intelligent Technology, Jinan, China); however, the in situ instantaneous local contact temperature during sliding was not monitored. A GCr15 bearing-steel ball (Zhejiang Zhongcheng Sliding Bearing Technology Co., Ltd, Quzhou, China) (Ø6 mm, HRC 65) was used as the counterbody and formed point contact with the QPQ-treated 40Cr disk; the as-treated surface condition was retained. Test parameters were: normal load, 10 N; wear-track diameter, 10 mm; rotational speed, 300 r/min; and duration, 30 min.

Corrosion resistance was evaluated in 3.5 wt.% NaCl using a three-electrode cell connected to a CHI electrochemical workstation (Shanghai Chenhua Instruments Co., Ltd, Shanghai, China). The sample (1.766 cm^2^ exposed area) served as the working electrode, with a saturated calomel electrode (SCE) as the reference and platinum as the counter electrode. Before testing, the open-circuit potential (OCP) was monitored. The sample was immersed for 30 min to allow OCP stabilization. Electrochemical impedance spectroscopy (EIS) was then performed from 10^−2^ to 10^5^ Hz using a 5 mV sinusoidal perturbation. After EIS, potentiodynamic polarization was conducted from OCP − 500 mV to OCP + 500 mV at a scan rate of 1 mV/s. Corrosion potential (***E_corr_***) and current density (***I_corr_***) were determined by Tafel fitting, and EIS spectra were analyzed by fitting to equivalent circuits in ZsimpWin (v3.60).

Salt-spray tests were conducted on QPQ-treated bars using an HL-90-BS chamber (KOMEG Technology Ind Co., Ltd, Guangdong, China), following the apparatus and temperature requirements of GB/T 10125-2021 [37]. A 3.5 wt.% NaCl solution was used as the spray medium. The chamber’s temperature was maintained at 35 °C, and the temperature of the saturator was set to 47 °C. The exposure time was 200 h. Samples were put on the same horizontal surface inside the chamber and arranged sequentially from left to right.

## 3. Results

### 3.1. Microstructure

**Figure 1:**
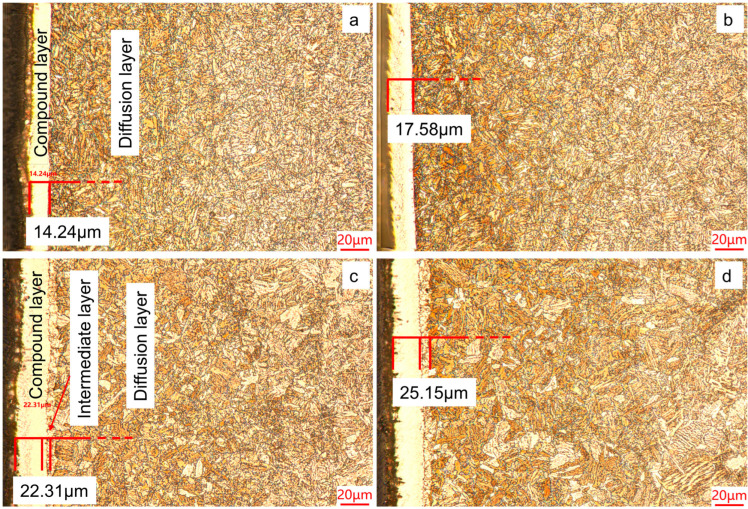
Metallographic structure of the cross-section after QPQ treatment. (**a**) 580 °C × 90 min; (**b**) 580 °C × 120 min; (**c**) 620 °C × 90 min; (**d**) 620 °C × 120 min.

Figure 1 shows cross-section microstructures of 40Cr steel after QPQ treatment under different conditions. All samples showed a typical layered QPQ structure, which includes a surface white compound layer and a lower diffusion layer containing acicular and granular nitrides. At 620 °C, the acicular nitrides became notably thicker; in addition, an intermediate transition layer was observed between the compound and diffusion layers. This transition layer is generally regarded as a buffer area that relates to a gradually reducing nitrogen concentration gradient. Blocky ferrite was also observed in the diffusion layer. These results indicate that the thickness of the compound layer is controlled by both temperature and holding time. When the temperature goes up from 580 °C to 620 °C, the compound layer’s thickness increases to more than 20 µm. In addition, prolonging holding time from 90 to 120 min also helps layer growth, with the thickness increasing by approximately 3 µm at both 580 °C and 620 °C.

**Figure 2:**
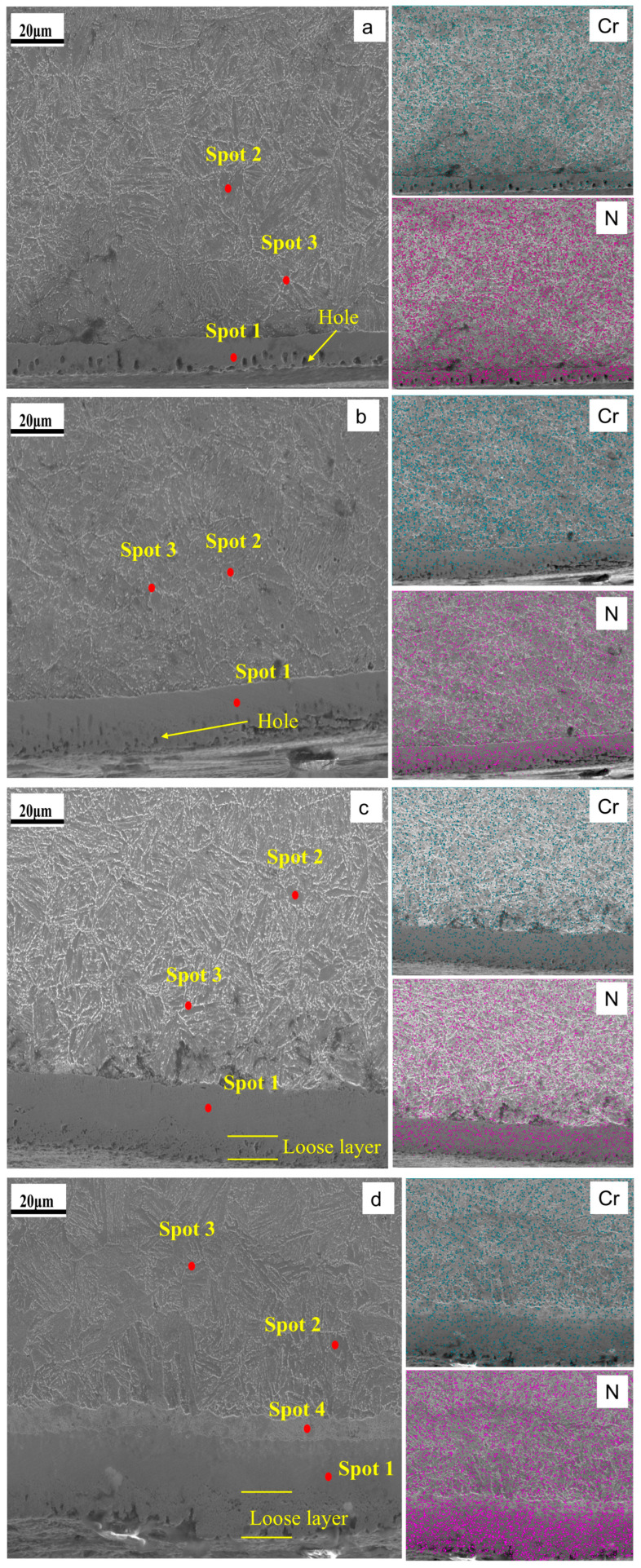
EDS elemental analysis results of different QPQ processes. (**a**) 580 °C × 90 min; (**b**) 580 °C × 120 min; (**c**) 620 °C × 90 min; (**d**) 620 °C × 120 min.

Figure 2 and Table 2 show cross-section SEM images and EDS results for samples treated under various QPQ conditions. Overall, the SEM observations were consistent with the optical micrographs: the modified layer comprised vein-like precipitates surrounded by blocky regions. The vein- and rod-like features are generally attributed to Fe_4_N [38]. Elemental maps showed that N was concentrated in the compound layer and decreased gradually toward the substrate. In contrast, Cr showed a highly heterogeneous distribution. Point EDS further showed that Cr content in regions adjacent to the vein-like features was higher than that within the veins. Notably, the 620 °C × 90 min condition showed a local decrease in N content within the compound layer, likely due to decomposition and volatilization of nitrogen-containing species.

**Figure 3:**
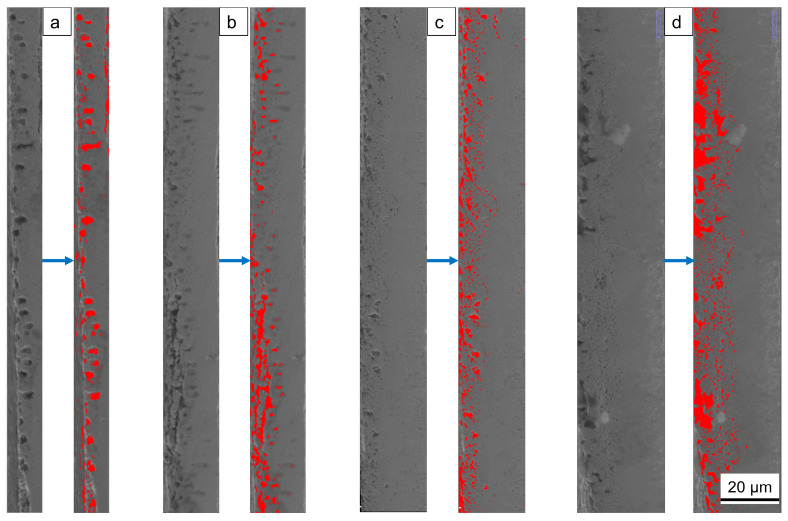
Schematic Comparison of Defect Areas. (**a**) 580 °C × 90 min; (**b**) 580 °C × 120 min; (**c**) 620 °C × 90 min; (**d**) 620 °C × 120 min.

Figure 3 magnifies the porosity and defects in the compound layer; Table 3 lists the calculation results. The compound layer exhibits two main defect types. At 580 °C, larger isolated pores dominate, and some pores begin to heal as processing time increases. At 620 °C, they transform into interconnected, sponge-like pores that expand inward with prolonged processing time. Since the pore size of these irregular, interconnected pores is difficult to quantify, this study uses defect fractions to characterize them.

### 3.2. XRD Phase Analysis

**Figure 4:**
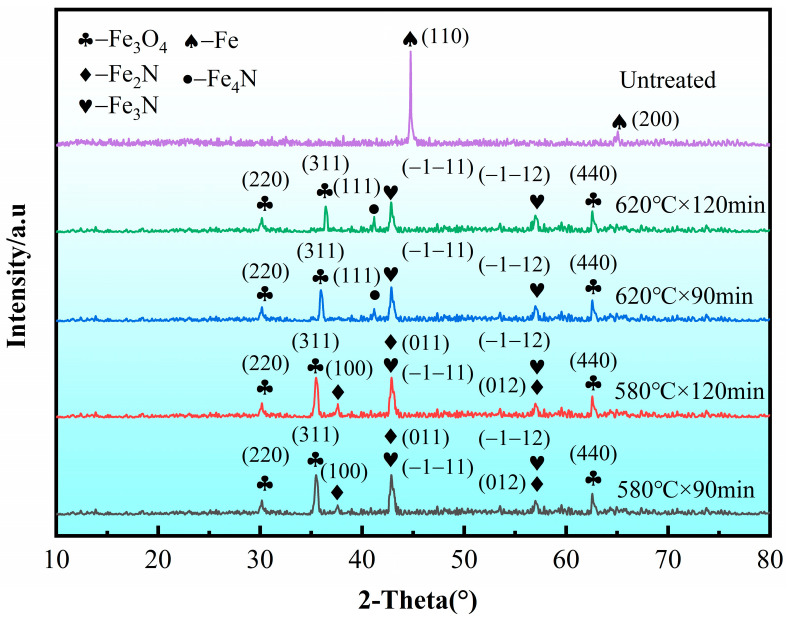
XRD test results of samples from different QPQ processes.

Figure 4 compares the XRD patterns of the samples before and after QPQ treatment. The XRD results indicate that the QPQ-treated layer mainly consists of Fe_3_O_4_ and iron nitrides, consistent with a typical oxide–nitride compound layer architecture. Compared with 620 °C, the Fe_3_O_4_ peaks at 580 °C were sharper and more intense, suggesting improved crystallinity and a more continuous oxide layer at 580 °C. Furthermore, at 620 °C, the Fe_2_N diffraction peaks were difficult to observe, and instead, Fe_4_N diffraction peaks were observed. This is because when the temperature exceeds 590 °C, ε-Fe_2–3_N gradually decomposes into γ′-Fe_4_N [39]. Meanwhile, γ′-Fe_4_N formed during ε-phase decomposition tends to nucleate and grow along the direction of nitrogen diffusion, producing a characteristic columnar morphology [40]. The reduced Fe_3_O_4_ intensity is attributed to oxide-film discontinuities, resulting from the formation of porous or loosely packed regions at elevated temperature.

### 3.3. Microhardness

**Figure 5:**
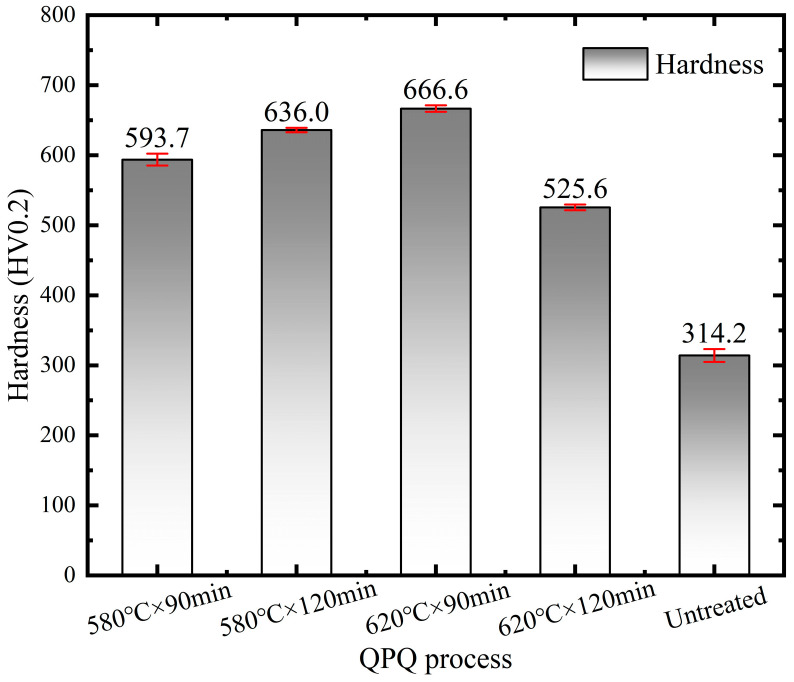
Surface hardness test results of different QPQ processes.

**Figure 6:**
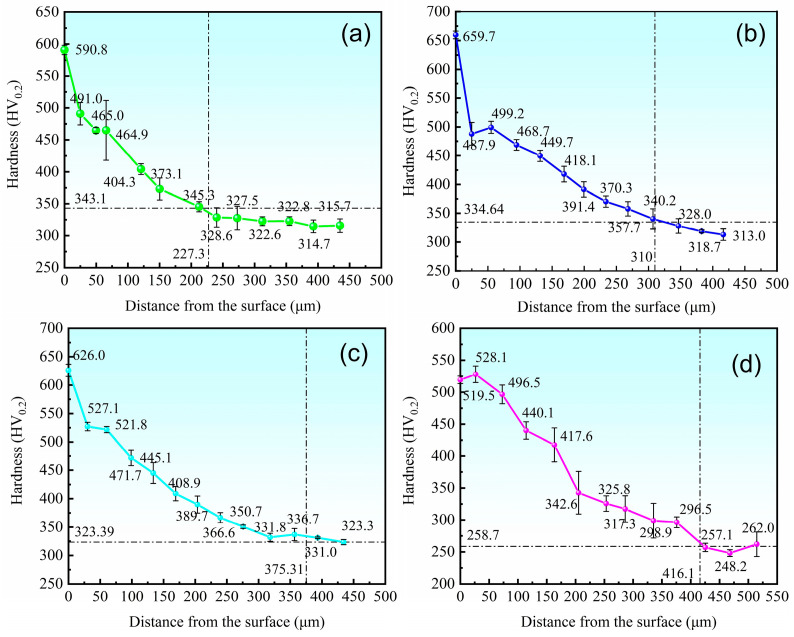
Cross-sectional gradient hardness test results of different QPQ processes. (**a**) 580 °C × 90 min; (**b**) 580 °C × 120 min; (**c**) 620 °C × 90 min; (**d**) 620 °C × 120 min.

Figure 5 and Figure 6 summarize the microhardness of samples after QPQ treatment. QPQ treatment markedly increased the surface hardness of 40Cr steel. Relative to the untreated state, surface hardness increased by 89% and 102% after treatment at 580 °C for 90 and 120 min, respectively. The maximum surface hardness occurred at 620 °C for 90 min (112% increase). Extending the holding time to 120 min reduced the increase to 67%. On the one hand, nitrogen atoms first enter the iron lattice as interstitial atoms, occupying octahedral interstitial sites and expanding the lattice. Consequently, residual stress increases due to the more expanded structure and high nitrogen supersaturation. Therefore, the surface hardness increases after nitriding treatments [41]. On the other hand, the high hardness of the ε and γ′ phases mainly accounts for the hardness increase. Nevertheless, as the ε phase transforms into the γ′ phase, the content of the lower hardness γ′ phase gradually increases [42]. Simultaneously, surface spongy porosity also increases. Ultimately, these factors together cause a decrease in hardness after treatment at 620 °C for 120 min.

The cross-section hardness profiles presented a typical surface-strengthening tendency: hardness reduced rapidly from the surface peak value along with depth increase and reached the substrate hardness level at about 350–450 µm. The steepest hardness gradient appeared in the near-surface area (0–50 µm), which mainly corresponds to the high-hardness compound layer and its transition zone. In the middle area (~50–300 µm), hardness reduced more slowly, which is consistent with the diffusion layer performance. GB/T 11354-2005 [43] defined diffusion-layer depth as the depth where hardness is 30 HV higher than the substrate value, as the dashed line in Figure 6 shows. The diffusion-layer depths were 227.3, 310.0, 375.3, and 416.1 µm for 580 °C × 90 min, 580 °C × 120 min, 620 °C × 90 min, and 620 °C × 120 min, respectively. Within the scope of this experiment, the diffusion layer depth exhibits an approximately linear relationship with increasing treatment temperature and holding time. This result indirectly shows that nitrogen activity increases and diffusion is accelerated. Hence, under the condition of 620 °C × 120 min, the hardness data showed larger error bars at several depth points. This fact suggests the occurrence of localized softening.

### 3.4. Friction and Wear Test

**Figure 7:**
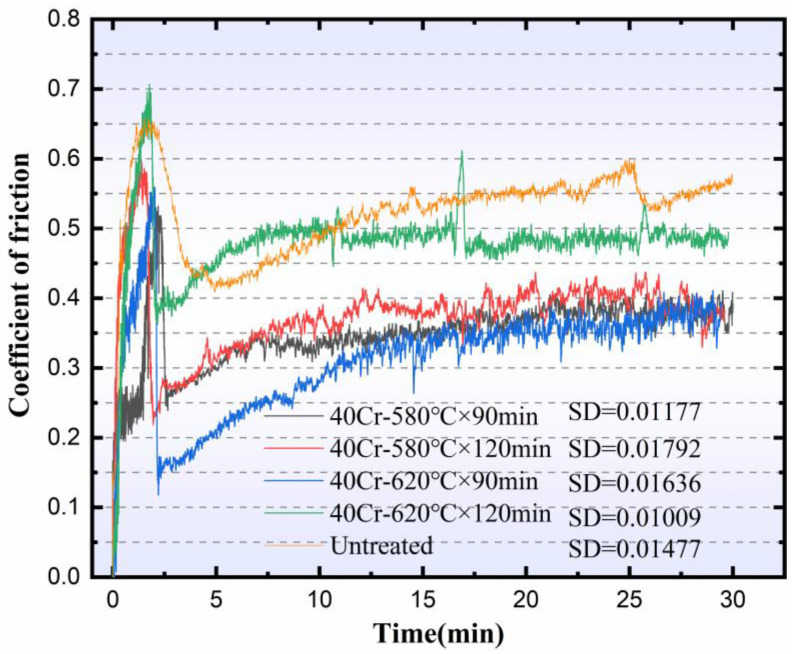
Tribological and wear test results of QPQ sample and original sample.

Figure 7 illustrates the temporal evolution of the coefficient of friction (COF) under dry sliding conditions for specimens subjected to varying QPQ treatment parameters. SD denotes the standard deviation of COF during the established steady-state interval. The associated quantitative data are presented in Table 4. All QPQ-treated specimens exhibited a distinct running-in regime at the onset of testing, characterized by a rapid increase and pronounced oscillations in COF. This was followed by a stabilized regime, indicative of a steady-state wear process. These observations suggest progressive removal of surface asperities, leading to a more stable, consistent tribological contact. In contrast, the untreated reference did not attain a steady-state COF during the entire test duration. Application of QPQ treatments resulted in a notable reduction in the average COF of 40Cr steel. The untreated substrate demonstrated the highest COF. Post-QPQ processing, COF values stabilized at 0.38, 0.39, 0.36, and 0.48 for the 580 °C × 90 min, 580 °C × 120 min, 620 °C × 90 min, and 620 °C × 120 min conditions, respectively. Karamis’s [44] research shows that, under dry sliding conditions, thicker composite layers, due to their porous and fragile structure, increase wear. This result is consistent with the wear behavior observed in this study (Figure 2 and Figure 7).

**Figure 8:**
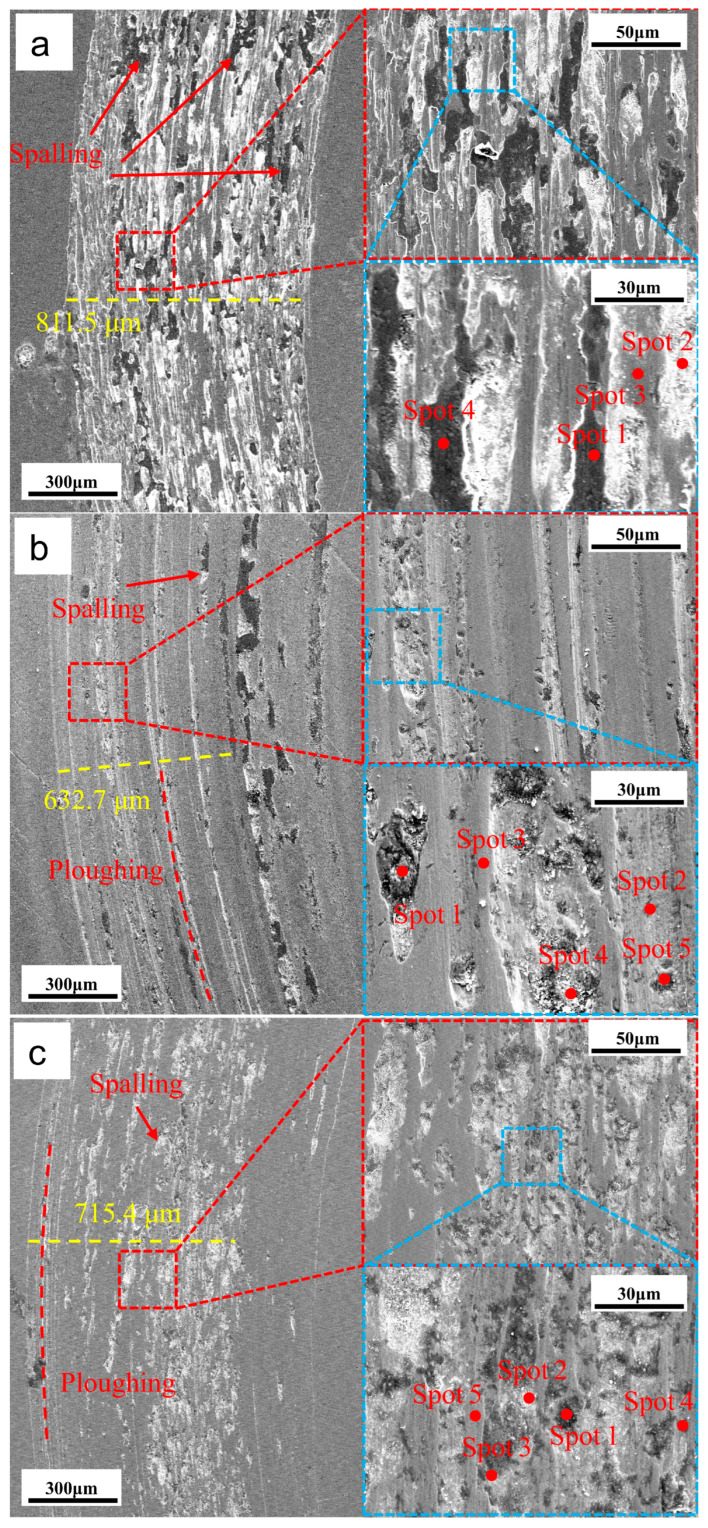
Wear morphology before and after QPQ treatment. (**a**) Untreated; (**b**) 580 °C × 120 min; (**c**) 620 °C × 120 min.

Figure 8 and Table 5 show the worn-surface morphology and changes in composition before and after QPQ treatment. The sample without treatment showed wide-range spalling and a highly uneven surface. Severe oxidative wear is indicated by high oxygen content in dark regions, Spots 1 and 4, with adhesive wear and fatigue spalling as dominant mechanisms. After the 580 °C × 120 min treatment, as shown in Figure 8b, the wear track became narrower and smoother, and continuous ploughing grooves became dominant on the surface. Spots 1 and 4, located in spalled pits, showed O enrichment; frictional heating likely promoted oxidation and led to oxide accumulation in these areas. Therefore, the dominant wear mechanism shifted to abrasive wear accompanied by mild oxidative wear. The wear track width also shows that the untreated sample has the largest, while the QPQ-treated sample has the smallest, due to the increased surface hardness. However, the sample treated at 620 °C for 120 min has a slightly wider wear track than the sample treated at 580 °C for 120 min, because the surface hardness is reduced.

At 620 °C, the wear tracks exhibit a mixed morphology of cracked grooves and noticeable spalling pits, but continuous ploughing marks become less prominent. These discontinuous pits increase surface roughness, disrupt the integrity of the oxide film, and thus hinder the formation of a continuous lubrication interface, thereby increasing the coefficient of friction. Furthermore, the debris falling from these spalling pits may promote three-body wear and exacerbate localized spalling.

### 3.5. Corrosion Resistance Test

**Figure 9:**
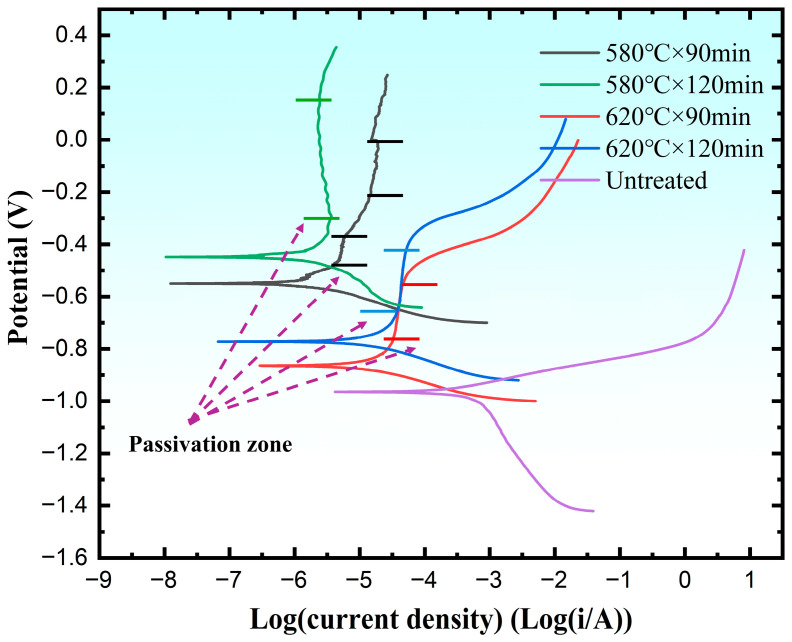
Polarization curves of QPQ sample and original sample.

To evaluate the influence of QPQ treatment on the corrosion resistance of 40Cr steel, potentiodynamic polarization curves were measured in a 3.5 weight-percent NaCl solution, as shown in Figure 9. Using the Tafel extrapolation method, corrosion potential, corrosion current density, and anodic and cathodic Tafel slopes were determined. The parameters obtained from this fitting procedure are presented in Table 6. Polarization resistance was also calculated using the Stern–Geary relationship [45]:

(1) $$R_{p}=\frac{b_{a}b_{c}}{2.303i_{corr}\left(b_{a}+b_{c}\right)},$$

where ***b_a_*** and ***b_c_*** are taken as absolute values.

Compared with the untreated sample, QPQ-treated samples showed an overall positive shift in ***E_corr_*** and a marked decrease in ***i_corr_***, indicating reduced corrosion tendency and a lower corrosion rate. Accordingly, ***R_p_*** increased from 179 Ω·cm^2^ for the substrate to 1610–24,500 Ω·cm^2^ after QPQ treatment. The percentage increases in ***R_p_*** for 580 °C × 90 min, 580 °C × 120 min, 620 °C × 90 min, and 620 °C × 120 min were 7386.03%, 13,587.15%, 799.44%, and 860.89%, respectively. These phenomena show stronger inhibition of interface charge transfer and slower total corrosion dynamics. When comparing processing conditions, samples treated at 580 °C exhibit lower ***i_corr_*** and higher ***R_p_*** than those treated at 620 °C, indicating better corrosion resistance. The passivation behavior in polarization curves suggests that a compact passive film forms during immersion. Because this film serves as a physical barrier that restricts electrolyte and substrate contact and inhibits anodic dissolution, it enhances the corrosion protection of 40Cr steel. At the same temperature, while the ***i_corr_*** values after prolonged holding are similar, considering both ***R_p_*** and the passivation zone width indicates that further extending the holding time improves the corrosion resistance of the 40Cr alloy.

**Figure 10:**
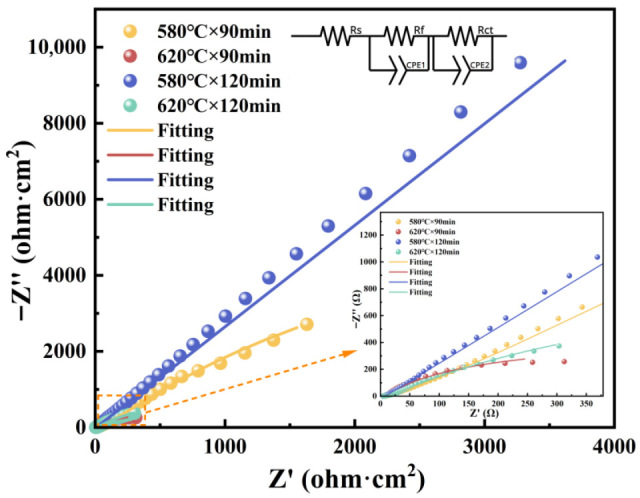
Nyquist plot of QPQ sample.

Figure 10 displays Nyquist plots of QPQ-processed samples in 3.5 wt.% NaCl solution, as well as the equivalent circuit used for fitting. In the circuit, Rs represents the solution resistance. A constant phase element (***CPE***) models non-ideal capacitive behavior. This is due to surface roughness, porosity, and compositional inhomogeneity. ***R_f_*** and ***R_ct_*** correspond to the film resistance and charge-transfer resistance, respectively. The equivalent circuit has two ***CPE***//***R*** elements in series: ***R_s_***–(***Q*_1-_**//***R_f_***)–(***Q*_2_**//***R_ct_***). This shows that two time constants control the impedance response. Specifically, (***Q*_1-_**//***R_f_***) represents the barrier effect of the surface film, (***Q*_2_**//***R_ct_***) describes the double-layer capacitance, and Faradaic charge transfer at the metal/electrolyte interface.

Figure 10 shows that impedance responses change clearly with processing parameters. This is seen in the different radii of capacitive arcs in the Nyquist plots. A larger arc radius indicates higher resistance to interfacial charge transfer and better corrosion resistance. The 580 °C × 120 min condition produced the largest arc radius and the optimal corrosion resistance. Next were 580 °C × 90 min, 620 °C × 120 min, and 620 °C × 90 min. The fitted parameters in Table 7 also show that both ***R_f_*** and ***R_ct_*** at 580 °C were higher than at 620 °C. The impedance results show that treatment at 580 °C offers more effective protection than at 620 °C.

**Figure 11:**
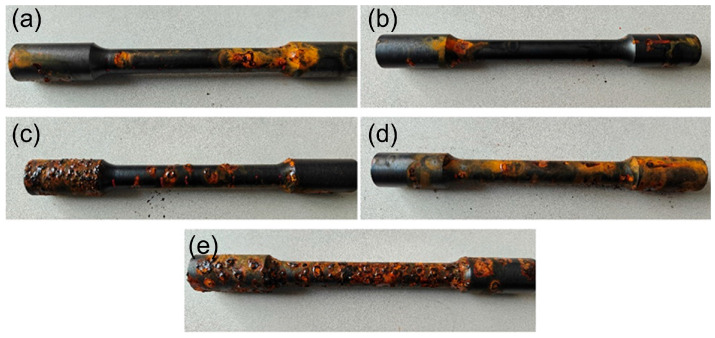
Salt spray test results before and after QPQ treatment. (**a**) 580 °C × 90 min; (**b**) 580 °C × 120 min; (**c**) 620 °C × 90 min; (**d**) 620 °C × 120 min; (**e**) Untreated.

Figure 11 shows the salt-spray corrosion morphologies of samples before and after QPQ treatment. After salt-spray exposure, the untreated sample exhibited extensive reddish-brown corrosion, indicating both general corrosion and pitting. In contrast, QPQ-treated samples largely retained an intact black surface film, with only localized rust spots, indicating improved corrosion resistance in the salt-spray environment.

Compared with different processing conditions, the sample treated at 580 °C showed less rust and a more dispersed rust distribution. Sample (b) presents the most even surface, with the fewest and smallest rust spots. In contrast, sample (a) has only a few localized rust marks, and its surface film remains mostly continuous. Samples treated at 620 °C display more localized corrosion and larger accumulations of corrosion products. Sample (c) exhibits more severe rusting and localized damage, with corrosion gathering at the shaft shoulder and other geometric discontinuities.

## 4. Discussion

The study shows that after QPQ treatment, all 40Cr steel samples form a layer of iron nitride compounds, with a Fe_3_O_4_ oxide film covering the surface (Figure 1 and Figure 4). This improved the hardness, friction properties, and corrosion resistance of the samples (Figure 5, Figure 7 and Figure 9). However, the microstructure and phase composition of this composite layer vary with QPQ temperature and holding time. At 620 °C, some ε-Fe_2–3_N transforms into γ′-Fe_4_N, forming a continuous sponge-like porous structure (Figure 2, Figure 3 and Figure 4). These defects and phase transitions affect the final surface properties. These defects and phase changes affect the final surface performance.

Based on this, the hardness test results can also be explained. The near-surface hardness of all QPQ-treated samples was significantly higher than that of the matrix, confirming the strengthening effect of the nitride-containing layer. However, in the samples treated at 620 °C × 120 min, the large hardness fluctuations and the presence of more spongy defects indicate that the local load-bearing capacity of the compound layer becomes uneven (Table 3, Figure 5 and Figure 6). One reasonable explanation is that the formation of spongy pores reduces the effective support area of the hardened surface and increases local stress concentration during indentation or sliding contact [46]. Moreover, the γ phase is usually 100 HK_0.05_ lower in hardness than the main nitride phase [42], which is also related to the presence of the γ phase in the XRD pattern at 620 °C.

The test results under dry friction conditions also showed a similar trend to the change in hardness. Compared with untreated 40Cr steel, the friction coefficients of all QPQ-treated samples were significantly reduced, indicating that the oxide-nitride layer effectively improved dry sliding performance. The wear mechanism changed from adhesive wear to abrasive wear, which was related to its higher surface hardness and the presence of a certain amount of γ′ phase with higher toughness [40]. However, even with the increased thickness of the composite layer, extending the treatment time at 620 °C to 120 min also increased the friction coefficient. This result indicates that the improvement in tribological properties is not determined by the thickness of the compound layer. It is more closely related to the expansion of defects in the near-surface region. With increased sponge-like porosity, pores become crack-initiation points [47]. At these locations, shear strength decreases and brittleness increases. The ε phase is more likely to undergo brittle spalling, which may exacerbate frictional wear [48,49]. This explanation is consistent with the pore morphology and wear surface morphology (Figure 3 and Figure 8). Under treatment at 620 °C for 120 min, the wear surface exhibits rougher grooves and localized spalling. Furthermore, the oxide film formed during post-oxidation can partially fill micro-pores and reduce surface roughness [50]. However, repeated rolling and grinding of hard debris at the contact interface disrupted the Fe_3_O_4_ lubricating layer, increased mechanical resistance, and raised COF.

The corrosion results showed that the surface integrity of both the oxide and compound layers was equally dependent on corrosion performance. All QPQ-treated samples exhibited higher corrosion potentials and lower corrosion current densities than untreated samples, confirming the protective effect of the post-oxidation nitriding and carburization layer (Figure 9, Table 6). Samples treated at 580 °C × 120 min showed higher polarization resistance, larger Nyquist arc radius, and higher ***R_f_*** and ***R_ct_*** values, indicating effective barrier properties against chlorine-containing solutions. In contrast, the two samples treated at 620 °C showed lower ***R_f_*** and ***R_ct_*** values (Figure 10, Table 6 and Table 7). These results are consistent with microstructural evidence that the compound layer with a lower defect percentage is more resistant to electrolyte penetration, while the formation of sponge-like pores weakens the continuity of the protective surface system. This is consistent with the findings of E.J. Mittemeijer and J. Kalucki [51,52]. This result can be explained by the fact that the transition from the ε phase to the γ′ phase may be accompanied by the redistribution and outward diffusion of nitrogen, which may promote the accumulation of local vacancies, denitrification, and eventually the formation of pores under overtreatment conditions [53]. Defects such as pores and microcracks can lower the local breakdown threshold and promote the initiation of pitting corrosion [54]. In addition, the subsequent oxidation reaction will form an oxide film on the surface, transforming the system from a single layer of nitride to a multilayer barrier layer composed of oxide films on the nitride surface. Electrochemical impedance spectroscopy (EIS) results (Figure 10, Table 7) support this view. When the Fe_3_O_4_ surface film is more homogeneous (***CPE*_1_** index is closer to 1), both film resistance and charge transfer resistance increase. Conversely, samples with higher defect density show lower film resistance and charge transfer resistance, indicating that the electrolyte is more easily penetrated and the compound layer has a poorer protective effect.

Overall, current research results indicate that both the ε and γ′ phases formed after QPQ treatment can increase surface hardness, modify the dry-sliding response, and enhance corrosion resistance under the present test conditions. However, prolonged high-temperature (e.g., 620 °C) treatment can lead to the transformation of the ε phase into the γ′ phase, which results in the formation of sponge-like pores and reduced surface density, thereby weakening surface properties. Despite this, several mechanism-related explanations in the current work remain inferential. The mechanical stability of the compound layer was primarily evaluated through hardness distribution curves rather than through direct tests of local elastic modulus, fracture resistance, or residual stress. Future research should combine nanoindentation with XRD-based residual stress analysis to verify the proposed link between porosity formation and load-bearing instability. Because the corrosion-related discussion depended mainly on electrochemical test data, depth-resolved XPS or GDOES would help clarify near-surface component gradients and their connection to protective film breakdown. In tribology, contact temperature affects oxidation during friction; therefore, monitoring temperature and analyzing frictional oxidation are valuable for comprehensively understanding wear mechanisms. Furthermore, the three-body wear mechanism proposed in this paper is mainly based on wear morphology and existing literature. As a logical next step, in-depth analysis of wear debris will be the focus of future research to further verify its accuracy.

## 5. Conclusions

The QPQ treatment formed an oxide–nitride composite layer on the surface of 40Cr steel, mainly composed of Fe_3_O_4_, ε-Fe_2–3_N, and γ′-Fe_4_N, together with a continuous diffusion layer. This improves the surface hardness and corrosion resistance of 40Cr steel and lowers its coefficient of friction under dry friction conditions.

At 580 °C, the surface layer was dominated by the ε phase, and defects were mainly present as relatively large, randomly distributed pores that tended to heal with prolonged holding time. At 620 °C, the ε phase progressively transformed into the γ′ phase, and the defects mainly appeared as continuously distributed, sponge-like pores that tended to extend inward with increasing treatment time.

Phase evolution and the generation of microdefects negatively affect the material’s surface properties. Under the condition of treatment at 620 °C for 120 min, on the one hand, they weaken the mechanical support of the surface, leading to a certain degree of reduction in the hardness of the matrix; on the other hand, due to the weakening of the surface’s resistance to deformation and the change in the contact state, the coefficient of friction increases under dry friction conditions. This microstructural change also reduces the surface film’s physical shielding effect, leading to lower film resistance and charge transfer resistance in EIS tests. Therefore, in this study, the sample treated at 580 °C for 120 min exhibited the best corrosion resistance.

## Figures and Tables

**Table 1:** QPQ Treatment Process.

QPQ Process	Quenching	Oil-Cooled	Tempering	Cooling	Nitriding	Oxidation
Process parameters	840 °C × 120 min	60 °C	600 °C × 120 min	Air-cooled	580 °C × 90 min	380 °C × 30 min
					580 °C × 120 min	
					620 °C × 90 min	
					620 °C × 120 min	

**Table 2:** EDS spot scan results for 40Cr.

QPQ Process	Element	Spot 1 (wt.%)	Spot 2 (wt.%)	Spot 3 (wt.%)	Spot 4 (wt.%)
580 °C × 90 min	N	6.93	4.47	1.50	None
	O	0.20	2.90	1.57	
	Fe	86.73	85.02	91.35	
	Cr	6.14	7.60	5.57	
580 °C × 120 min	N	7.26	2.18	4.93	None
	O	0.64	1.57	2.63	
	Fe	85.57	91.54	88.38	
	Cr	6.53	4.72	4.06	
620 °C × 90 min	N	4.50	3.68	6.08	None
	O	0.17	2.26	2.18	
	Fe	86.22	86.80	88.52	
	Cr	9.11	7.27	3.22	
620 °C × 120 min	N	9.47	1.98	2.95	5.54
	O	1.06	1.46	3.04	2.88
	Fe	86.49	90.32	89.18	86.87
	Cr	2.98	6.24	4.82	4.70

**Table 3:** Defect area and proportion for different QPQ processes.

QPQ Process	Total Area of Compound Layer/μm^2^	Defect Area/μm^2^	Defect Area ± SD/%
580 °C × 90 min	1108.59	87.36	7.88 ± 0.22
580 °C × 120 min	1693.52	132.09	7.80 ± 0.17
620 °C × 90 min	2153.85	177.04	8.22 ± 0.30
620 °C × 120 min	2708.33	348.29	12.86 ± 0.54

**Table 4:** The COF between the QPQ sample and the original sample.

QPQ Process	COF	Standard Deviation
580 °C × 90 min	0.38	0.01177
580 °C × 120 min	0.39	0.01792
620 °C × 90 min	0.36	0.01636
620 °C × 120 min	0.48	0.01009
Untreated	0.56	0.01477

**Table 5:** EDS analysis results of wear morphology before and after QPQ treatment.

Process	Element	Spot 1 wt.%	Spot 2 wt.%	Spot 3 wt.%	Spot 4 wt.%	Spot 5 wt.%
Untreated	N	0.79	1.47	1.13	0.84	None
	O	22.59	9.04	4.1	26.5	
	Cr	1.03	1.25	1.62	1.18	
	Fe	75.59	88.24	93.16	71.48	
580 °C × 120 min	N	2.22	1.62	1.16	1.05	1.46
	O	27.7	1.96	6.07	14.92	2.75
	Cr	1.44	2.11	1.5	1.39	1.42
	Fe	68.64	94.31	91.27	82.64	94.37
620 °C × 120 min	N	3.38	3.07	2.01	6.64	1.43
	O	25.48	19.13	3.89	1.69	1.2
	Cr	1.11	1.48	1.15	1.47	1.39
	Fe	70.03	76.32	92.96	90.2	95.98

**Table 6:** Polarization curve fitting results of the original QPQ sample.

Process	*E_corr_*/V	*i_corr_*/A·cm^−2^	Anode Slope (*b_a_*)	Cathode Slope (*b_c_*)	Polarization Resistor (*R_p_*)/Ω·cm^2^
580 °C × 90 min	−0.5372	1.79 × 10^−6^	0.1755	−0.08036	13,400
580 °C × 120 min	−0.4325	1.80 × 10^−6^	0.3232	−0.1481	24,500
620 °C × 90 min	−0.8711	1.63 × 10−^5^	0.3512	−0.07279	1610
620 °C × 120 min	−0.7769	1.64 × 10^−5^	0.3074	−0.08266	1720
Untreated	−0.9426	4.51 × 10^−4^	0.4878	−0.2991	179

**Table 7:** EIS fitting data.

Parameter	580 °C × 90 min	580 °C × 120 min	620 °C × 90 min	620 °C × 120 min
***R_s_***/Ω·cm^2^	8.74	5.02	4.63	5.15
***CPE*_1_**-Y_0_	3.10 × 10^−3^	8.22 × 10^−4^	2.61 × 10^−2^	2.39 × 10^−2^
***CPE*_1_**-n	0.745	0.771	0.334	0.338
***R_f_***/Ω·cm^2^	56.2	72.6	5.68	19.1
***CPE*_2_**-Y_0_	8.31 × 10^−2^	2.00 × 10^−3^	1.83 × 10^−2^	1.41 × 10^−2^
***CPE*_2_**-n	0.445	0.666	0.830	0.715
***R_ct_***/Ω·cm^2^	1.16 × 10^4^	2.91 × 10^4^	7.89 × 10^2^	2.53 × 10^3^
***χ*** ^2^	8.79 × 10^−4^	9.40 × 10^−4^	1.82 × 10^−3^	1.43 × 10^−3^

## Data Availability

The original contributions presented in this study are included in the article. Further inquiries can be directed to the corresponding authors.
